# Correlation of hepcidin and serum ferritin levels in thalassemia patients at Chiang Mai University Hospital

**DOI:** 10.1042/BSR20203352

**Published:** 2021-02-16

**Authors:** Adisak Tantiworawit, Sujaree Khemakapasiddhi, Thanawat Rattanathammethee, Sasinee Hantrakool, Chatree Chai-Adisaksopha, Ekarat Rattarittamrong, Lalita Norasetthada, Pimlak Charoenkwan, Somdet Srichairatanakool, Kanda Fanhchaksai

**Affiliations:** 1 Division of Hematology, Department of Internal Medicine, Faculty of Medicine, Chiang Mai University, Chiang Mai 50200, Thailand; 2 Division of Hematology and Oncology, Department of Pediatrics, Faculty of Medicine, Chiang Mai University, Chiang Mai 50200, Thailand; 3 Department of Biochemistry, Faculty of Medicine, Chiang Mai University, Chiang Mai 50200, Thailand

**Keywords:** β-thalassemia major, β-thalassemia/Hb E, ferritin, Hb H disease, hepcidin

## Abstract

Hepcidin is a key iron-regulatory hormone, the production of which is controlled by iron stores, inflammation, hypoxia and erythropoiesis. The regulation of iron by hepcidin is of clinical importance in thalassemia patients in which anemia occurs along with iron overload. The present study aimed to evaluate the correlation between serum hepcidin and ferritin levels in thalassemia patients. This cross-sectional study investigated 64 patients with thalassemia; 16 β-thalassemia major (BTM), 31 β-thalassemia/hemoglobin (Hb) E (BE), and 17 Hb H + AE Bart’s disease (Hb H + AE Bart’s). The levels of serum hepcidin and ferritin, and Hb of the three groups were measured. The median values of serum ferritin and Hb were significantly different among the three groups, whereas serum hepcidin values were not observed to be significantly different. The correlation of the serum hepcidin and ferritin levels was not statistically significant in any of the three groups of thalassemia patients with BTM, BE, or Hb H + AE Bart’s (r = −0.141, 0.065 and −0.016, respectively). In conclusion, no statistically significant correlations were observed between serum hepcidin with any variables including serum ferritin, Hb, age, labile plasma iron (LPI), and number of blood transfusion units among the three groups of thalassemia patients. Likely, the regulation of hepcidin in thalassemia patients is affected more by erythropoietic activity than iron storage.

## Introduction

Thalassemia is the most commonly inherited type of hemolytic anemia in Thailand and is caused by a globin chain defect. In incidences of thalassemia, red blood cells (RBCs) do not function properly and survive for shorter periods of time. Consequently, anemia and other related complications can occur. In Thailand, degrees of frequency are 20–30% for the α-thalassemia trait and Hb E trait, 3–9% for the β-thalassemia trait, and 1% for thalassemia disease [1,2]. Patients with β-thalassemia display an absence or a reduced level of β-globin chain synthesis leading to a reduction in Hb in the RBC and ultimately, anemia [3,4]. The basic therapy for β-thalassemia major (BTM) is regular blood transfusions resulting in iron overload, whereas β-thalassemia intermedia (BTI) is a less severe form of anemia than BTM.

Excess iron in vital organs is known to cause impaired organ function and increased rates of morbidity and mortality [5]. In transfusion-dependent thalassemia (TDT) patients, iron overload mainly occurs as a result of transfusions. In comparison, iron overload in cases of non-transfusion-dependent thalassemia (NTDT) can occur from increased intestinal absorption despite receiving occasional transfusions [3,6]. To assess body iron in thalassemia patients, serum ferritin is a key component of a relatively easy and practical approach; however, the levels are influenced by infection and inflammation. Consequently, a diagnosis of iron overload using serum ferritin would require serial measurements [7,8].

Iron absorption in humans is regulated by the combined influences of the body’s erythropoietic demand for iron, tissue oxygenation, and the body’s iron stores [10]. Hepcidin, a 25-amino acid peptide produced by hepatocytes, is the key hormone involved in the control of iron homeostasis at the point of convergence of the erythroid and the regulated stores of iron [9,10]. The peptide then binds to the iron export channel ferroportin, thereby inducing its internalization and degradation and leading to the inhibition of cellular iron efflux [9,11]. The overexpression of serum hepcidin levels results in iron restricted anemia [12], while low serum hepcidin levels are known to increase intestinal iron absorption and decrease iron stores in macrophages leading to iron overload [13]. Many studies have shown that low serum hepcidin levels in β-thalassemia patients can lead to increased iron absorption levels and iron overload [9,14–17]. The regulation of iron by hepcidin is of clinical importance in thalassemia patients as anemia often occurs along with iron overload. Hepcidin as a therapeutic target might help the management of iron overload in thalassemia patients [15,18]. In the present study, we purposed to determine the correlation between serum hepcidin and ferritin levels in Thai thalassemia patients.

## Materials and methods

### Patients and study design

This was a cross-sectional study involving patients at Chiang Mai University Hospital, Faculty of Medicine, Chiang Mai University, Chiang Mai, Thailand from October 2013 to October 2014. The study included 64 patients diagnosed with thalassemia as follows; 16 BTM, 31 β-thalassemia/Hb E (BE), and 17 Hb H+AE Bart’s diseases. All patients provided their written informed consent after the study has been approved. The eligibility criteria included the following: being diagnosed with BTM, BE, or hemoglobin H + AE Bart’s disease (Hb H + AE Bart’s) with an age of at least 15 years old. Patients were excluded if they had received a prior transfusion of packed red cells within 20 days or had experienced a clinical infection. Medical records and Hb typing results obtained by high performance liquid chromatography (HPLC) analysis were reviewed in terms of the type of thalassemia and the patient’s history of blood transfusions for either TDT or NTDT subjects. The NTDT group was defined as patients that had received blood transfusions less than three times per year. The patients were instructed to withhold iron-chelating agents at least 72 h before blood samples were taken. Venous blood (5 ml) was withdrawn from each participant from which 2 ml was added to a tube containing ethylenediamine tetraacetic acid (EDTA) anticoagulant and the other one (3 ml) was added to the tubes containing the clot activator. The clotted blood was centrifuged and separated immediately to a new tube for analysis of biochemical parameters.

### Analysis of hematological parameters

A complete blood count (CBC) test was performed immediately with peripheral blood smears, and the samples were then stained with Wright–Giemsa stain. Hb was measured according to the sodium lauryl sulfate (SLS)-Hb method using an XN1000 SYSMEX machine.

### Measurement of serum ferritin

Serum ferritin was measured using electro-chemiluminescence immunoassay (ECLIA) according to the manufacturer's instructions (Cobas e801 immunoassay analyzer, Cobas®, Roche Diagnosis GmbH, Mannheim, Germany).

### Measurement of serum hepcidin

Serum hepcidin concentration was measured using a competitive enzyme-linked immunosorbent assay (cELISA) kit (Catalog No. CEb979Hu, Cloud-Clone Corp., Uscn Life Science Inc., Wuhan, P.R. China) according to the manufacturer’s instructions [19,20].

### Quantification of serum non-transferrin bound iron

Non-transferrin bound iron (NTBI) was quantified using the nitrilotriacetate (NTA) chelation/HPLC method established by Singh et al. [21]. In assay, serum was incubated with a weak chelator NTA (80 mM at a final concentration) in (3-(N-morpholino)propanesulfonic acid (MOPS) buffer, pH 7.0 for 30 min at room temperature to produce Fe^3+^–(NTA)_2_ complex. Afterward, the complex was filtered through a membrane (NanoSep®, 10-kDa cutoff, polysulfone type; Pall Life Sciences, Ann Arbor, MI, U.S.A.) and analyzed using a non-metallic HPLC system. NTBI was fractionated on a glass analytical column (ChromSep-ODS1, 100 mm × 3.0 mm, 5-μm particle size), eluted isocratically with mobile-phase solvent (3 mM CP22 in 19% acetonitrile/MOPS pH 7.0) at a flow rate of 1.0 ml/min and optical density (OD) was monitored online at 450 nm using a flow cell detector (SpecMonitor2300; LDC Milton-Roy Inc., Riviera Beach, FL, U.S.A.). Data analysis was conducted with BDS software (BarSpec Ltd., Rehovot, Israel). NTBI concentration represented by Fe^3+^-(CP22)_3_ peak area was determined from a calibration curve constructed from Fe^3+^–(NTA)_2_ in 80 mM NTA (0–16 μM).

### Measurement of labile plasma iron

In principle, redox-active labile plasma iron (LPI) can convert non-fluorescent dihydrorhodamine (DHR) into oxidized form rhodamine (R), resulting in an increase in fluorescence intensity (FI). Serum (20 μl) was incubated with the DHR solution containing ascorbic acid at 37°C for 30 min and kinetics of increasing FI was followed immediately for 40 min, with readings every 2 min using a spectrofluorometer (λ_excitation_ 485 nm, λ_emission_ 538 nm). Slope (ΔFI/min) was determined and plotted against the reaction time between 15 and 40 min. A calibration curve was constructed by plotting the slope against standard ferrous ammonium sulfate (FAS) solution (0–20 μM). Difference in rate of DHR oxidation represents a component of redox active LPI in the serum, which the LPI concentration was calculated from the calibration curve [22,23].

### Statistical analysis

Statistical analyses were performed with SPSS version 23. Descriptive data were reported as mean ± standard derivation (SD) values, as well as median and interquartile range (IQR) values. The clinical data were compared among the three groups of thalassemia patients using Chi-square test for the categorical data and Kruskal–Wallis Test for the non-parametric data. The degree of correlation for hepcidin and quantitative variables in each of the three groups was measured using the Spearman’s Correlation Test. *P*-value <0.05 is considered statistically significant.

## Results

### Demographic data

Sixty-four patients (60.9% females) with a median age of 29 years (17–58 years) and mean Hb value of 7.3 g/dl (4.8–9.6 g/dl) were included in the present study. Patients had been diagnosed with BE (48.4%), Hb H + AE Bart’s (26.6%), BTM (25.0%), respectively. The 64 patients were identified as TDT (48.4%). Approximately 47% of the patients had received a splenectomy. Patients with iron overload (75.0%) received iron-chelating agents (65.7%) including deferiprone (32.8%), desferrioxamine (26.6%), and deferasirox (6.3%), respectively. The prevalence of co-morbidities included subclinical hypothyroidism (15.6%), elevated liver enzymes without viral/drug induced hepatitis (9.0%), impaired fasting glucose (6.3%), primary hypothyroidism (6.3%), osteoporosis (3.1%), diabetes mellitus (1.6%), and pulmonary hypertension (1.6%), respectively (Table 1).

**Table 1:** Demographic data, type of thalassemia, splenectomy, Hb levels, transfusion dependency, iron overload and chelations, and co-morbidity in 64 Thai thalassemia cases

Characteristics	Number of patients (%)
**Female**	39 (60.9)
**Type of thalassemia**	
BTM	16 (25.0)
BE	31 (48.4)
Hb H disease+ AE Bart’s	17 (26.6)
**Splenectomy**	30 (46.9)
** TDT**	31 (48.4)
**Iron overload**	48 (75.0)
**Iron chelations**	
None	22 (34.3)
Desferrioxamine	17 (26.6)
Deferiprone	21 (32.8)
Deferasirox	4 (6.3)
**Co-morbidities**	
Subclinical hypothyroidism	10 (15.6)
Elevated liver enzymes	6 (9.0)
Impaired fasting glucose	4 (6.3)
Primary hypothyroidism	4 (6.3)
Osteoporosis	2 (3.1)
Diabetes mellitus	1 (1.6)
Pulmonary hypertension	1 (1.6)

### Clinical and laboratory data

The mean ages of the thalassemia patients with BTM, BE, and Hb H+ AE Bart’s were 24.5 ± 1.2, 31.2 ± 1.9, and 39.4 ± 2.8 years, respectively. Notably, there were significant differences among the three groups (*P*=0.001). The distribution of gender among the three groups revealed no statistically significant differences (*P*=0.841). The percentages of patients requiring regular blood transfusion were significantly different among the three groups (*P*<0.001). The median (IQR) of the number of blood transfusion units in the three groups of thalassemia patients with BTM, BE, or Hb H + AE Bart’s disease was 18.5 (10), 3 (14) and 0 (0), respectively. The number of blood transfusion units was significantly different among the three groups of thalassemia patients (*P*<0.001). The percentages of patients with iron overload were significantly different among the three group (*P*=0.01). The median levels of serum hepcidin and NTBI were not found to be significantly different among the three groups (*P*=0.224 and *P*=0.427, respectively). The median levels of serum ferritin, Hb, and LPI were significantly different among the three groups of thalassemia patients (*P*=0.002, 0.017, and 0.004, respectively) (Table 2).

**Table 2:** Comparison of clinical and laboratory data of thalassemia patients

Variables	BTM (*n*=16)	BE (*n*=31)	Thalassemia Hb H+ AE Bart’s (*n*=17)	*P*-value
**Age (years)**	24.5 ± 1.2	31.2 ± 1.9	39.4 ± 2.8	0.001^*^
**Gender (male/female)**	7/9	11/20	7/10	0.841
**Transfusion dependence**	16/16 (100%)	16/31 (51.6%)	1/17 (5.9%)	<0.001^*^
**Number of blood transfusion units**				
**Median (IQR)**	18.5 (10)	3 (14)	0 (0)	<0.001^*^
**Range**	12–31	0–30	0–4	
**Iron overload**	16/16 (100%)	22/31 (71.0%)	10/17 (58.8%)	0.01^*^
**Serum hepcidin (ng/ml) [normal male 29–254 ng/ml, normal female 17–286 ng/ml] [19]**				
**Median (IQR)**	40.69 (23.50)	44.95 (18.03)	45.12 (22.26)	0.224
**Range**	23.18–74.75	24.28–76.29	29.22–72.58	
**Serum ferritin (ng/ml)**				
**Median (IQR)**	2105 (1977)	1234 (927)	967 (986)	0.002^*^
**Range**	933–7245	208–6255	164–3815	
**Hb (g/dl)**				
**Median (IQR)**	7.1 (0.9)	7.4 (1.9)	8.3 (1.9)	0.017^*^
**Range**	6.0–8.4	5.5–9.3	4.6–9.6	
**NTBI (μM)**				
Median (IQR)	0.45 (0.16)	0.48 (0.29)	0.55 (0.31)	0.427
Range	0.39–3.56	−0.24–13.43	0.37–0.85	
**LPI (μM)**				
**Median (IQR)**	4.76 (2.2)	4.81 (8.2)	1.6 (3.3)	0.004^*^
**Range**	1.36–14.29	−2.03–13.49	−3.03–6.83	

Data are expressed in numbers or mean ± SD. *P< 0.05 is considered statistically significant.

### Correlation of serum hepcidin with serum ferritin and demographic variables

**Figure 1:**
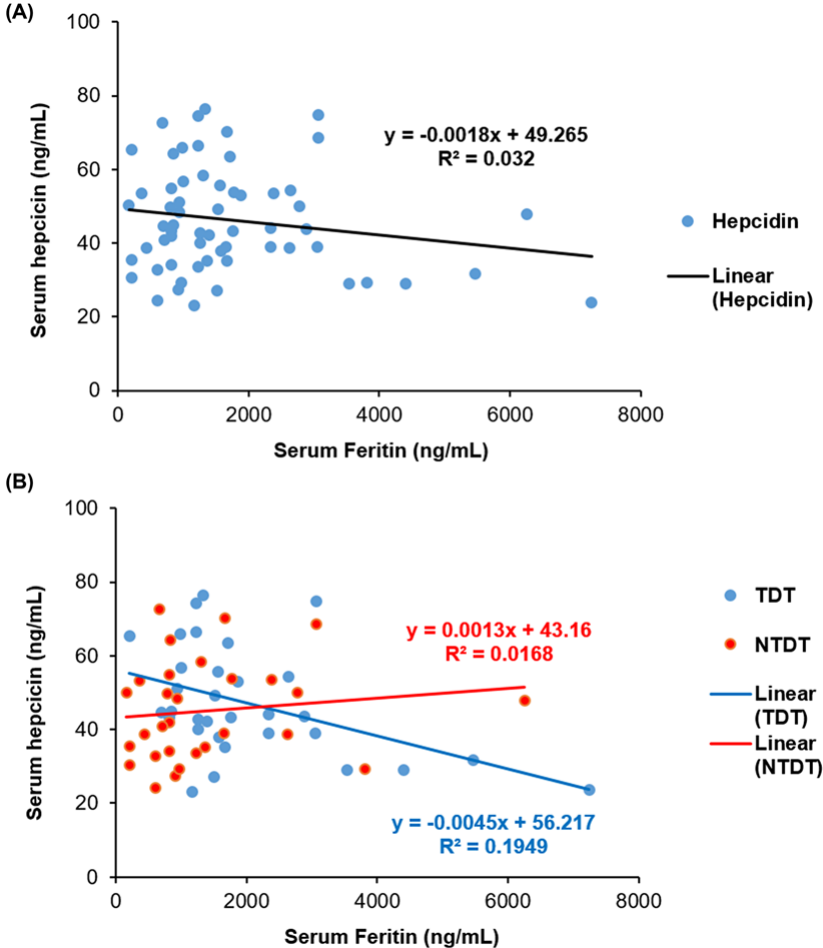
The correlation between serum hepcidin and serum ferritin (**A**) Linear correlation between serum hepcidin and serum ferritin levels in all thalassemia patients (R^2^ = 0.032). (**B**) Linear correlation between serum hepcidin and serum ferritin levels in TDT (R^2^ = 0.1949) and NTDT (R^2^ = 0.0168) patients.

No statistically significant correlation was observed between serum hepcidin and serum ferritin levels in thalassemia patients (r_s_ = −0.046, *P*=0.727). The subdivision between TDT and NTDT, the correlation between serum hepcidin and serum ferritin levels was not statistically significantly different in TDT (r_s_ = −0.330, *P*=0.075) and NTDT (r_s_ = 0.112, *P*=0.550). Linear correlation between serum hepcidin and serum ferritin levels in all thalassemia patients (R^2^ = 0.032) (Figure 1A), TDT (R^2^ = 0.1949) (Figure 1B), and NTDT (R^2^ = 0.0168) (Figure 1B), respectively. Correlations between serum hepcidin values and laboratory and demographic variables in the subgroup analysis of the thalassemia patients are shown in Table 3. No statistically significant correlations were observed between serum hepcidin values with regard to all variables including serum ferritin, Hb, age, LPI, and number of blood transfusion units among the three groups of the thalassemia patients (Table 3). Correlations of serum hepcidin and ferritin levels were not statistically significant in any of the three groups of thalassemia patients with BTM (r_s_ = −0.141, *P*=0.602), BE (r_s_ = 0.065, *P*=0.739), or Hb H+AE Bart’s (r_s_ = −0.016, *P*=0.953) (Table 3).

**Table 3:** Correlation between serum hepcidin values and laboratory and demographic variables in thalassemia patients

Group	Serum ferritin (ng/ml)		Hb (g/dl)		Age (y)		LPI (μM)		Number of blood transfusion units	
	r_s_	*P*-value	r_s_	*P*-value	r_s_	*P*-value	r_s_	*P*-value	r_s_	*P*-value
**BTM**	−0.141	0.602	−0.007	0.978	−0.186	0.491	−0.106	0.696	−0.115	0.670
**BE**	0.065	0.739	−0.308	0.104	−0.148	0.442	−0.020	0.917	0.084	0.663
**Thalassemia Hb H+ AE Bart’s**	−0.016	0.953	0.057	0.834	−0.346	0.189	0.034	0.901	−0.393	0.132

Abbreviation: r_s_, Spearman’s rho correlation coefficient.

## Discussion

Our data showed the results of hepcidin in Thai thalassemia patients. The median serum hepcidin levels were not observed to be significantly different among the three groups of thalassemia patients; however, the median serum hepcidin levels in subjects with BTM were lower than in the patients diagnosed with BE and Hb H + AE Bart’s diseases. The serum hepcidin levels in the present study were within the 5–95% reference range reported in healthy adult male 112 ng/ml [29–254 ng/ml] and female 65 ng/ml [17–286 ng/ml]. The median serum hepcidin levels in the present study were lower than those reported in healthy adults. [19]. Multiple previous studies have reported lower serum hepcidin levels in β-thalassemia patients with iron overload [9,14–17]. One *in vitro* study found that HepG2 cells treated with the serum from nine BTM patients resulted in down-regulated hepcidin levels [16]. This outcome supports the claim that hepcidin down-regulation induced by thalassemia can lead to iron overload. Another *in vivo* model revealed that the iron metabolism gene (*Hfe*) effectively down-regulated hepcidin in a mouse model of BTI (th^3/+^), while increasing incidences of anemia and iron overload [14]. On the other hand, th^3/+^ mice with increased hepcidin levels as a result of overexpression of hepcidin gene (*Hamp1*) showed limited iron overload and improved circumstances of anemia [15].

The median levels of serum ferritin and Hb were significant among the three groups of thalassemia patients, which were consistent with the findings of previous studies [24,25]. The percentages of patients requiring regular blood transfusion were significantly different among the three groups. In addition, iron overload may have occurred from regular blood transfusions in BTM subjects when compared with patients with BE and Hb H + AE Bart's diseases as serum ferritin levels were significantly higher in the BTM subjects.

Our study results revealed no significant correlations between serum hepcidin and ferritin levels among the three groups of thalassemia patients. The findings of our study were in accordance with those of previous studies, wherein no significant correlation of serum hepcidin and ferritin levels was observed in BTM and BTI patents in southern Iran [24], in children with BTM in India [25], in pediatric polytransfused (>20 transfusions) patients with BTM [26], and in multitransfused patients with BTM [27]. Notably, serum hepcidin levels decreased due to high erythroid signals [24]. The ferritin levels were observed to be significantly higher in patients with BTM than in those with BE and Hb H + AE Bart’s diseases, respectively. This condition of iron overload could have resulted from regular blood transfusions in BTM subjects when compared with BTI patients.

Our data showed that the NTBI values were not different among the three groups of thalassemia and the LPI values were not different between BTM and BE but significantly lower in Hb H + AE Bart’s. This may be explained by the high percentages of iron overload in all groups. A previous study showed the mean serum iron of the thalassemic patients (*n*=25) was 38.5 ± 7.1 pmol/l with a total iron binding capacity (TIBC) saturation of 85%, in comparison, the mean serum iron of the normal controls (*n*=50) was 17.8 ± 3.0 pmol/l with 29% saturation of the TIBC [28]. Our study has a limitation that transferrin saturation was not measured. The values should have added to the explanation of the high NTBI values in our patients.

## Conclusions

No statistically significant correlations were observed between serum hepcidin with any variables including serum ferritin, Hb, age, LPI, and number of blood transfusion units among the three groups of thalassemia patients. Likely, the regulation of hepcidin in β-thalassemia patients was more affected by erythropoietic activity than iron overload. Importantly, one limitation of the present study was that the erythropoietic activity was not measured.

## Data Availability

The data used to support the findings of the present study are included within the article.

## Abbreviations

BE: β-thalassemia/Hb E
BTI: β-thalassemia intermedia
BTM: β-thalassemia major
DHR: dihydrorhodamine
FI: fluorescence intensity
Hb H + AE Bart’s: hemoglobin H + AE Bart’s disease
Hb: hemoglobin
HPLC: high performance liquid chromatography
IQR: interquartile range
LPI: labile plasma iron
MOPS: 3-(N-morpholino)propanesulfonic acid
NTA: nitrilotriacetate
NTBI: non-transferrin bound iron
NTDT: non-transfusion-dependent thalassemia
R: rhodamine
RBC: red blood cell
TDT: transfusion-dependent thalassemia
